# An adjusted bed net coverage indicator with estimations for 23 African countries

**DOI:** 10.1186/1475-2875-12-457

**Published:** 2013-12-20

**Authors:** Dieter Vanderelst, Niko Speybroeck

**Affiliations:** 1 University Antwerp Faculty of Applied Economics Prinsstraat 13, Antwerp 2000, Belgium; 2 Faculté de Santé Publique et Institut de recherche Santé et Société, Universite Catholique de Louvain, Place de l’Université, 1, Louvain-la-Neuve B-1348, Belgium

## Abstract

**Background:**

Many studies have assessed the level of bed net coverage in populations at risk of malaria infection. These revealed large variations in bed net use across countries, regions and social strata. Such studies are often aimed at identifying populations with low access to bed nets that should be prioritized in future interventions. However, often spatial differences in malaria endemicity are not taken into account. By ignoring variability in malaria endemicity, these studies prioritize populations with little access to bed nets, even if these happen to live in low endemicity areas. Conversely, populations living in regions with high malaria endemicity will receive a lower priority once a seizable proportion is protected by bed nets. Adequately assigning priorities requires accounting for both the current level of bed net coverage and the local malaria endemicity. Indeed, as shown here for 23 African countries, there is no correlation between the level of bed net coverage and the level of malaria endemicity in a region. Therefore, the need for future interventions can not be assessed based on current bed net coverage alone. This paper proposes the Adjusted Bed net Coverage (ABC) statistic as a measure taking into account both local malaria endemicity and the level of bed net coverage. The measure allows setting priorities for future interventions taking into account both local malaria endemicity and bed net coverage.

**Methods:**

A mathematical formulation of the ABC as a weighted difference of bed net coverage and malaria endemicity is presented. The formulation is parameterized based on a model of malaria epidemiology (Smith *et al*. Trends Parasitol 25:511-516, 2009). By parameterizing the ABC based on this model, the ABC as used in this paper is proxy for the steady-state malaria burden given the current level of bed net coverage. Data on the bed net coverage in under five year olds and malaria endemicity in 23 Sub-Saharan countries is used to show that the ABC prioritizes different populations than the level of bed net coverage by itself. Data from the following countries was used: Angola, Burkina Faso, Burundi, Cameroon, Congo Democratic Republic, Ethiopia, Ghana, Guinea, Kenya, Liberia, Madagascar, Malawi, Mali, Mozambique, Namibia, Nigeria, Rwanda, Senegal, Sierra Leone, Tanzania, Uganda, Zambia and Zimbabwe. The priority order given by the ABC and the bed net coverage are compared at the countries’ level, the first level administrative divisions and for five different wealth quintiles.

**Results:**

Both at national level and at the level of the administrative divisions the ABC suggests a different priority order for selecting countries and divisions for future interventions. When taking into account malaria endemicity, measures assessing equality in access to bed nets across wealth quintiles, such as slopes of inequality, are prone to change. This suggests that when assessing inequality in access to bed nets one should take into account the local malaria endemicity for populations from different wealth quintiles.

**Conclusion:**

Accounting for malaria endemicity highlights different countries, regions and socio-economic strata for future intervention than the bed net coverage by itself. Therefore, care should be taken to factor out any effects of local malaria endemicity in assessing bed net coverage and in prioritizing populations for further scale-up of bed net coverage. The ABC is proposed as a simple means to do this that is derived from an existing model of malaria epidemiology.

## Background

Geospatial techniques have been used to estimate the distribution of malaria vectors and malaria burden [1]. Recently, these efforts have culminated in a high-resolution map of the global malaria endemicity [2,3] confirming the existence of large between and within country differences in malaria burden, vector prevalence and endemicity. The most effective way to prevent malaria infection is using an insecticide-treated bed net [4]. In spite of the large-scale programmes that have been undertaken to distribute nets [5], nets are not uniformly distributed across the population at risk. Bed net ownership varies among countries, regions and social strata [5,6].

The spatial variation in both malaria endemicity and bed net coverage strongly suggests that some populations are at greater risk than others. In particular, populations living in regions of high malaria endemicity but with low levels of bed net coverage are at high risk. Indeed, if bed net coverage and malaria endemicity are not strongly correlated, they are two independent components of the level of protection against malaria. In this case, the level of malaria endemicity should be taken into account when determining the level of protection or when setting priorities for bed net distribution as for populations with similar bed net coverage the level of local malaria endemicity will vary considerably.

Recent data on bed net coverage (obtained through surveys) and a map of global malaria endemicity [2,3] allow investigating the relation between bed net coverage and malaria endemicity for many countries. In this paper, data from 23 sub-Saharan countries are used to propose an Adjusted Bed net Coverage (ABC) statistic taking into account both bed net coverage and malaria endemicity. It is shown how the ABC can be used in identifying populations characterized by a low level of bed net usage living in regions with malaria endemicity. Such populations would be prime targets for scaling up bed net coverage through additional programmes.

## Methods

### Bed net coverage and malaria endemicity

**Figure 1:**
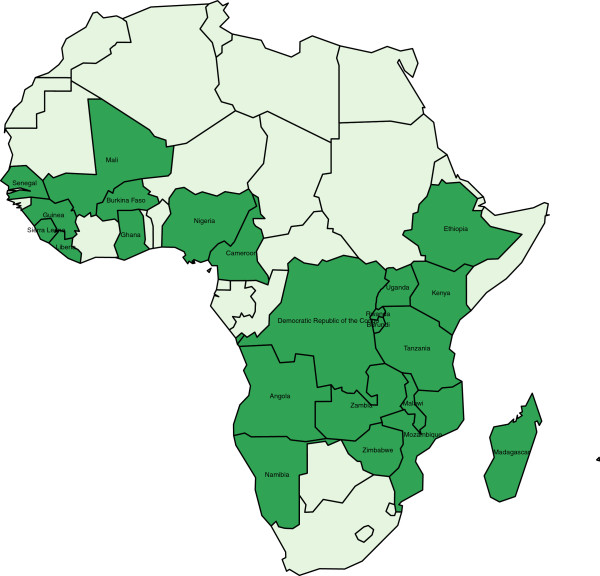
Map of the 23 countries included in the current study.

Data on bed net use were obtained from the MEASURE Demographic and Health Surveys (DHS). In this paper, the level of bed net coverage in children under five is used. All Demographic and Health Surveys (DHS) and Malaria Indicators Surveys (MIS) conducted in sub-Saharan Africa were included provided data were available in June 2013. If more than one survey was conducted in a particular country, the most recent survey was used provided it contained all necessary variables. Some surveys or countries had to be omitted from the analysis as the available data were deemed too old or because variables critical to the current study were missing (see Additional file 1 for more details about the omitted countries and surveys). In total, data from 23 countries were analysed in the current study (see Figure 1 and Table 1).

**Table 1:** Listing of the 23 countries included in the study, the year in which the survey started and ended, the number of children sampled and the average level of bed net coverage

	**Country**	**Start**	**End**	**N**	**Bed net**
		**year**	**year**		**coverage**
1	Angola	2011	2011	7714	0.25
2	Burkina Faso	2010	2010	13716	0.43
3	Burundi	2010	2011	7231	0.42
4	Cameroon	2011	2011	10734	0.10
5	Congo Democratic	2007	2007	7987	0.05
	Republic				
6	Ethiopia	1997	1997	9002	0.01
7	Ghana	2008	2008	2794	0.37
8	Guinea	2005	2005	5641	0.01
9	Kenya	2008	2009	5706	0.43
10	Liberia	2011	2011	3149	0.33
11	Madagascar	2011	2011	6101	0.73
12	Malawi	2012	2012	2218	0.54
13	Mali	2006	2006	12437	0.23
14	Mozambique	2011	2011	10291	0.33
15	Namibia	2006	2007	4858	0.09
16	Nigeria	2010	2010	5379	0.26
17	Rwanda	2010	2011	8484	0.65
18	Senegal	2010	2011	11633	0.32
19	Sierra Leone	2008	2008	5043	0.24
20	Tanzania	2011	2012	8289	0.66
21	Uganda	2011	2011	7355	0.39
22	Zambia	2007	2007	5844	0.26
23	Zimbabwe	2010	2011	5203	0.09

The DHS selects clusters of households to be surveyed in a two-stage cluster sampling design. The GPS coordinates of these clusters are recorded using GPS receivers. To ensure respondent confidentiality, the latitude/longitude positions are displaced for all surveys. Urban clusters are displaced by maximally 2 kilometres and rural clusters by maximally 5 kilometres. Moreover, 1% of the rural clusters are displaced by up to 10 km. In sum, for over 99% of the clusters the provided GPS coordinates are correct up to at least 5 km.

For each cluster, the level of *P. falciparum* endemicity in 2010 as provided by the Malaria Atlas Project was extracted at the recorded GPS location. More specifically, the age-standardized *P. falciparum* parasite rate (PfPR2-10) was extracted describing the estimated proportion of 2–10 year olds in the general population that are infected with *P. falciparum* at any one time, averaged over the 12 months of 2010.

The first-level administrative divisions for all included countries were downloaded from the GADM database of Global Administrative Areas. The level of bed net coverage and malaria endemicity for each first-level administrative division was determined by taking the weighted mean of the bed net coverage and malaria endemicity for all clusters in the division using the sample weights provided by the DHS.

Evaluating the differences in bed net coverage across different social strata is done based on the wealth quintiles provided by the DHS. For each sampled household, the DHS constructs a wealth index using easy-to-collect data on a household’s ownership of selected assets, such as televisions and bicycles; materials used for housing construction; and types of water access and sanitation facilities. The wealth quintiles divide the sampled households into five different levels of wealth. In many instances, the wealth index or quintile has been shown to be an important factor in a household’s access to healthcare with richer households having usually better access to provisions.

### The Adjusted Bed net Coverage (ABC)

In this section, the rationale behind the Adjusted Bed net Coverage statistic (ABC) is clarified by means of four hypothetical regions with a different bed net coverage and malaria endemicity as listed in Table 2. When considering the bed net coverage, region D is nearly optimally protected (bed net coverage is 0.9) while region A is almost not protected at all (bed net coverage is 0.1). This seems to imply that region A should be highly prioritized over regions D in future interventions to increase its bed net coverage. However, when taking into account malaria endemicity, it becomes clear that the difference in malaria risk between regions A and D is not as large as the bed net coverage would let one to belief. The low and high malaria endemicity in regions A and D respectively are likely to compensate the difference in bed net coverage between the regions and the malaria risk in region D is arguably as high as in region A. In spite of the difference in bed net coverage, future interventions aimed at increasing or maintaining the bed net coverage in region D might be as pressing as interventions to increase bed net coverage in region A. This illustrates that bed net coverage is not a sufficient measure to prioritize regions for intervention.

**Table 2:** Four hypothetical regions with different levels of bed net coverage and malaria endemicity

	**Coverage**	**Endemicity**	**Adjusted**	**Coverage**	**ABC**
				**rank**	**rank**
Region A	0.10	0.10	0.55	1	3
Region B	0.30	0.70	0.26	2	1
Region C	0.70	0.30	0.68	3	4
Region D	0.90	0.90	0.39	4	2

The third column gives the ABC calculated using Equation 4. The fourth and fifth column give the priority order as deduced from the level of bed net coverage and the ABC respectively. See text for details.

Quantifying the risk in regions A-D and ranking them requires a model of malaria epidemiology that allows an informed weighting of both parameters. Indeed, epidemiological models quantifying the effects of bed nets, akin to the one proposed by Smith *et al*. [7], can be used to determine how the two parameters should be weighted in order to get an estimate of the malaria risk (at the equilibrium state, see Smith *et al*. [7] for details) for a given malaria endemicity and achieved level of bed net coverage. Using the ABC statistic that is proposed in the next section of the paper, reveals a different priority order for the four regions than when ordering the regions according to their level of bed net coverage alone (see Table 2).

In sum, the example listed in Table 2 illustrates that (1) both bed net coverage and malaria endemicity should be taken into account when determining the malaria risk of a given population and (2) the weight assigned to the parameters needs to be based on epidemiological models. In the next section of the paper, the ABC statistic is derived that is a weighted combination of both parameters.

### Mathematical definition of the Adjusted Bed net Coverage

The ABC assigns populations a value between 0 (no protection) and 1 (complete protection) and is calculated as the weighted difference of the malaria endemicity and bed net coverage. In the following, the formulation of the statistic is presented and it is shown how the weighting used here relates to a model of the reduction in malaria endemicity proposed by Smith *et al*. [7].

**Figure 2:**
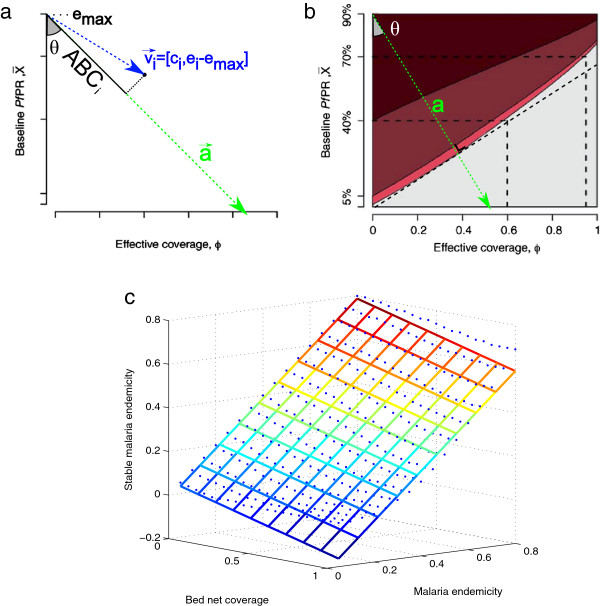
**Geometrical definition of the adjusted bed net coverage.(a)** Geometric definition of the ABC statistic. Populations *i* characterized by bed net coverage *c*_*i*_ and malaria endemicity *e*_*i*_ are projected onto a vector $\vec{a}$. This vector is to be chosen such that projection *ABC*_*i*_ of region *i* is higher for lower values of *c*_*i*_ and lower for higher values of *e*_*i*_. By parameterizing the vector using angle *θ* and *e*_*max*_ the correspondence with external statistics of protection can be optimized. In this paper, *θ* is chosen such that *ABC*_*i*_ corresponds with the reduction of malaria endemicity for a given endemicity and bed net coverage as predicted by the model of Smith *et al*. [7]. **(b)** Figure altered from Smith *et al*. [7]. For a set of benchmark parameters, the resulting malaria endemicity as a function of baseline endemicity and bed net coverage. The colours represent different endemicity levels (dark red, >40%; red, 5%-40%; pink, 1%-5%; and gray, <1%). In this paper, *θ* was choosen such that the $\vec{a}$ is orthogonal to the boundary of the area for which the final malaria endemicity is lower than 1%. **(c)** Results of fitting a linear model to data of Smith *et al*. [7]. The blue dots represent the predicted final malaria endemicity for a program that scaled-up the bed net coverage from 0 to a specific target level at the end of five years. The grid represents the result of a linear fit of the data. A linear model could fit the data very well (*R*^2^=0.97). The regression function was *E*_*stable*_=−0.0163−0.1971×*C*+1.0047×*E*. Although the fitted data is not the data displayed in Figure 2 and used to parameterize the ABC for the purpose of the current paper, it shows that the predictions of Smith *et al*. [7] can fitted adequately using a linear model.

Let $\vec{a}$ be the vector defined in Figure 2a and given in Equation (1).

(1) $$\vec{a}=\;[\;-\cos\theta ,\sin\theta ]$$

The *ABC*_*i*_ for population *i* is obtained by projecting population *i* with bed net coverage *c*_*i*_ and endemicity *e*_*i*_ onto vector $\vec{a}$. This results in a higher ABC being assigned to populations with a lower bed net coverage and a higher endemicity. To be able to project population *i* onto $\vec{a}$, the vector $\vec{v}$ is defined based on *c*_*i*_ and *e*_*i*_ as follows (see also Figure 2a),

(2) $$\vec{v_{i}}=\;[\;c_{i},e_{i}-e_{m}]$$

Projecting $\vec{v_{i}}$ on $\vec{a}$ results in a new vector with norm *ABC*_*i*_ giving the Adjusted Bed net statistic for population *i* (Equation 3). Note that the denominator in Equation 3 normalizes *ABC*_*i*_ to assume values between 0 ans 1.

(3) $${\text{ABC}}_{i}=\frac{\sin\theta \times c_{i}-\mathrm{cos\theta}\times (e_{i}-e_{\text{max}})}{\sin\theta +\mathrm{cos\theta}\times e_{\text{max}}}$$

As can be seen from Equations 1-3, the projection depends on a variable *e*_*max*_ and the angle *θ*. *e*_*max*_ is the level of malaria endemicity one wishes to associate with the minimum level of protection (i.e. if *c*_*i*_=0 and *e*_*i*_=*e*_*max*_ then *ABC*_*i*_=0). The angle *θ* is a parameter that controls the weighting of bed net coverage and malaria endemicity in determining the ABC. Higher values of *θ* assign more relative weight to the level of malaria endemicity and vice versa. For *θ*=0°, *ABC*_*i*_=*e*_*i*_. When *θ*=90°, *ABC*_*i*_=*c*_*i*_.

Selecting the value of *θ* can be done based on a priori assumptions on the relative importance of bed net coverage and endemicity in protection against malaria infection. However, as argued in the previous section, it is preferably based on malaria transmission and control models. Smith *et al*. [7] propose a model predicting the malaria burden based on bed net coverage and endemicity (Figure 2b). The prediction of their model allows to deduce the relative (numeric) importance of both bed net coverage and endemicity for the resulting malaria burden. As can be seen in Figure 2b, malaria burden rises more rapidly as function of endemicity than it decreases as a function of bed net coverage. Choosing $\vec{a}$ as perpendicular to one of the isocontours as shown in 2b results in an estimate for *θ* of about 34°. Therefore, in the current paper, *θ* was set to 34°. The parameter *e*_*max*_ was set to 0.9 as the data presented by Smith *et al*. [7] did not cover higher levels of endemicity. Furthermore, this was well above any of malaria endemicity of the household clusters included in this paper. By parameterizing Equation 3 based on the model of Smith *et al*. [7], ABC can be seen as proxy for the steady-state malaria burden given the current level of bed net coverage. Filling in the values for *θ* and *e*_*max*_ in Equation 3, results in the following equation (which will be used in the remainder of the paper) with *c*_*i*_ the level of bed net coverage in under five year olds and *e*_*i*_ the *PfPR*_2−10_ as provided by the Malaria Atlas Project.

(4) $${\text{ABC}}_{i}=0.57+0.43\cdot c_{i}-0.63\cdot e_{i}$$

Equation 4 shows that the ABC assumes that level of protection is a linear function of both bed net coverage and malaria endemicity (with a larger weight for malaria endemicity). Inspecting Figure 2b suggests that a linear function provides a good approximation of the model of Smith *et al*. [7]. The authors do not provide the numerical data used in creating the plot in Figure 2b but do provide numerical results for the simulated changes in *PfPR* for an ITN programme that scales-up bed net coverage for a period of five years. It was found that a linear model could fit these data very well (*R*^2^=0.97) assigning a larger weight to malaria endemicity than to bed net coverage (see Figure 2c).

In the Results section, the correlation between the bed net coverage and the ABC will be assessed using the Spearman Rank Correlation (SRC). The SRC assesses how well the ordering based on bed net coverage of different administrative divisions, countries and sub populations is retained by the ABC. Therefore, the SRC gives an indication of the difference in priority order assigned to administrative divisions, countries and sub-populations by the two statistics.

## Results

### Independence of malaria endemicity and bed net coverage

**Figure 3:**
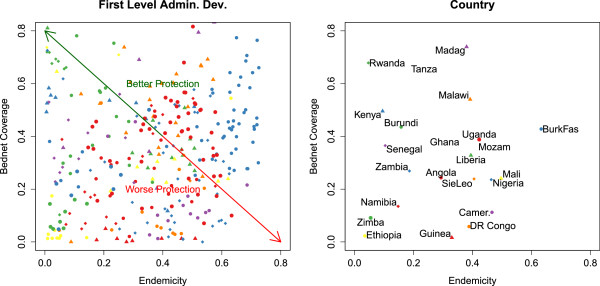
**Scatterplot of malaria endemicity and the level of bed net coverage in the 23 countries included in the study.** Left: Scatterplot of malaria endemicity and the level of bed net coverage in the first level administrative divisions of the countries included in the study. The colours and shapes of the markers correspond to those of the countries in the right panel. The red and the green arrow indicate the relevant dimension for the protection of a population: division in the left top corner of the plot are well protected. These are characterized by low levels of endemicity and high levels of bed net coverage. Conversely, divisions in the lower right hand corner are badly protected as the level of bed net coverage is low while experiencing high levels of malaria endemicity. The ABC statistic assigns populations a value between 0 and 1 along this axis. Right: idem, for countries. Country names have been abbreviated as needed.

Figure 3, shows the level of bed net coverage in under five year olds in each of the 23 countries as a function of the average level of malaria endemicity. This plot illustrates the absence of a strong correlation between these measures. Neither for the first level administrative divisions [ *SRC*:*r*=−0.04,*p*=0.84] nor at the level of the countries [ *SRC*:*r*=−0.04,*p*=0.84] a significant correlation was found between bed net coverage and malaria endemicity.

The absence of correlation between bed net coverage and malaria endemicity (Figure 3), indicates that bed net coverage and malaria endemicity are statistically independent components of protection against malaria infection. This was corroborated by estimating the mutual information between both variables. An empirical estimation of the entropy [8] yielded 3.13 bits and 3.25 bits for bed net coverage and the malaria endemicity in the first level administrative divisions respectively. As the data was binned into 10 intervals these values approach the maximum entropy value of 3.32 bits (*log*_2_(10)≈3.32). The mutual information between both variables was about 0.04 bits confirming the statistical independence of the variables.

The independence of both variables indicates that neither the level of bed net coverage nor the endemicity by itself is sufficient to identify populations with high priority for intervention. Indeed, a given bed net coverage is equally likely to be found in regions with any level of malaria endemicity. This justifies the construction of an ABC which allows taking into account both the level of bed net coverage and malaria endemicity in a region or population.

### ABC for countries and first level divisions

**Figure 4:**
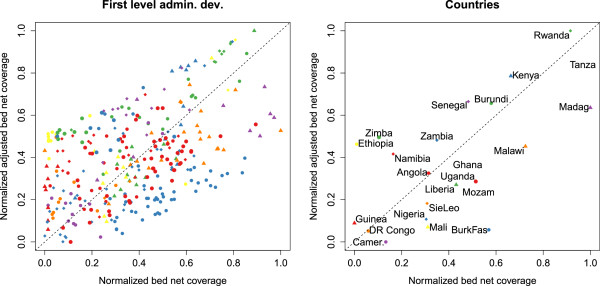
**Comparison of the ranking of countries and divisions by bed net coverage and ABC.** For this plot both statistics have been normalized by scaling them to between 0 and 1. This allows evaluating the differences in ranking of the divisions and countries by both statistics. Left: comparison between bed net coverage and ABC for the first level administrative divisions. Data points above the diagonal indicate divisions that rank higher on the ABC than on the bed net coverage statistic. Divisions below the diagonal rank lower on the ABC than on the bed net coverage. Right: same for the countries. The shape and colour of the markers in the right plot matches those in the left plot. For example, the administrative divisions of Malawi are represented by a orange triangle in the left plot. Country names have been abbreviated as needed.

The bed net coverage and ABC correlate significantly but not very strongly, both at the level of the administrative divisions [ *SRC*:*r*=0.58,*p*<0.01] and at the level of the countries [ *SRC*:*r*=0.65,*p*<0.01]. Indeed, Figure 4 shows that ordering countries according to ABC alters the priority order of first level administrative divisions and countries with respect to that obtained by using bed net coverage. For example, Zimbabwe having fairly low bed net coverage rates is better protected when considering the ABC. On the other hand, Malawi, having a high level of bed net coverage has a lower ABC. Other examples are Burkina Faso and Mali which rank lowly on the ABC.

**Figure 5:**
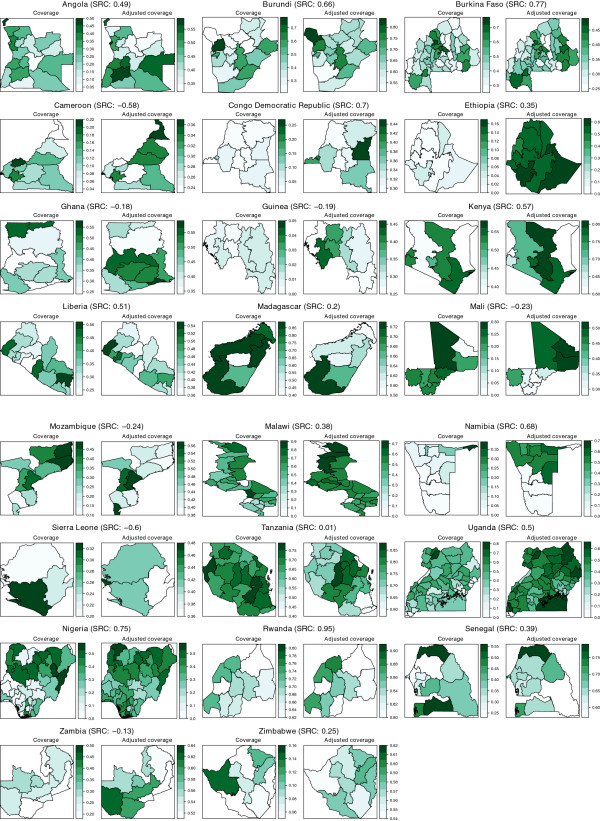
**Maps of the bed net coverage and ABC in the 23 countries included in the study.** Note that the aspect ratio of the maps has been set to 1:1. The Spearman rank correlation between the bed net coverage and ABC for each country is noted between brackets.

Within countries, the correlation between bed net coverage and ABC of the divisions varies considerably (Figure 5). In some countries, the SRC was very high (e.g. Rwanda, 0.95). For others, the correlations was about zero (e.g. Tanzania, 0.01) or negative (e.g. Cameroon, -0.57). The comparison between the divisional bed net coverage and ABC for all countries is shown by means of maps in Figure 5.

### Slopes of inequality

**Figure 6:**
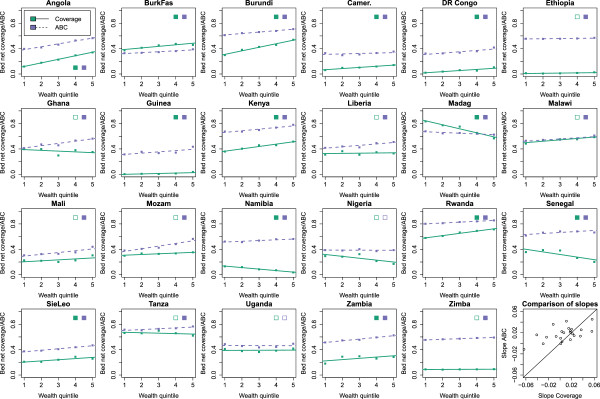
**Slopes of inequalities resulting from bed net coverage and the ABC for all countries.** The markers denote the bed net coverage (green) and ABC (purple) for the five wealth quintiles. The lines give the corresponding regression lines and slopes. The bottom right plot shows a scatterplot of both types of slopes. The statistical significance of the slopes, i.e. *p*<0.01, is indicated by the filled or open squares inside each panel. A filled square indicates a slope significantly different from 0 for the corresponding statistic.

Indicators of socio-economic inequalities of bed net coverage are prone to change as well when using the ABC (Figure 6). The slope of inequality was calculated for each country by regressing either the level of bed net coverage or ABC on the five wealth quintiles. A positive slope indicates that higher wealth quintiles have more bed nets. The slopes obtained by using the bed net coverage were larger than the slopes obtained using the ABC [paired t-test, *t*(22)=−2.09,*p*<0.05]. In addition, the variance of slopes obtained using the ABC was lower than that for the bed net coverage slopes [ *F*(22,22)=4.40,*p*<0.01]. This indicates that ABC slopes revealed a more consistent tendency for richer households to be better protected across countries. Moreover, while the slopes obtained through both measures correlated significantly [ *SRC*:*r*=0.57,*p*<0.01] substantial differences in slopes were observed for some countries. For example, in Liberia using the ABC results in a positive slope indicating better protection for richer children while the bed net coverage slope showed no statistical difference in protection between children from richer and poorer households. In other countries, including Namibia and Senegal, the sign of the slope changes with the indicator.

## Discussion

In a world with limited resources, allocating budgets should be done in an informed and justified way [9]. Smith *et al*. [7] argued that intervention planning should be based on the expected reduction in malaria burden. These authors provide a model of the expected malaria burden depending both on the endemicity and the achieved bed net coverage. A similar approach, based on the *P. falciparum* basic reproductive number under control, was proposed by Gething *et al*. [10]. The ABC statistic can be considered as a simple proxy to these suggestions as it allows taking into account both malaria endemicity and bed net coverage. Indeed, by parameterizing Equation 3 based on the model of Smith *et al*. [7] (i.e. *θ*=34°), the ABC is a linear approximation of the steady-state malaria burden given the current level of bed net coverage. Nevertheless, it can be calculated without resorting to a detailed epidemiological model. Indeed, the ABC only requires the current bed net coverage to be known. The malaria endemicity as used in this paper can be freely and simply obtained from the Malaria Atlas Project. In line with the suggestions by Smith *et al*. [7] and Gething *et al*. [10], it is proposed here to use the ABC statistic when setting priorities for intervention. Indeed, recently Omumbo *et al*. [9] showed that many sub-Saharan countries have access to at least one type of risk map to support the planning of interventions. However, only very few countries actually used this information to specify tailored sub-national? intervention plans or resource allocation. The ABC could be a simple standardized tool to facilitate the use of risk data in the planning of interventions thereby increasing the success of investments in malaria control.

Ultimately, the ABC as presented in the paper is a linear function derived from reference 8 (Equation 4). However, introducing the ABC using geometrical functions and providing Equation 3 allows for re-parameterization of the projection of the populations onto the relevant dimension in the endemicity/bed net coverage plane.

Logically any measure of malaria burden could be used to prioritize populations for interventions. Both epidemiological measures (e.g. level of infection) or clinical (e.g. mortality) could be used. However, the model of Smith *et al*. [7] takes data that is readily available from the Malaria Atlas Project (malaria endemicity) and the DHS (bed net coverage). Therefore, the data for calculating the ABC used here are freely available for a large number of countries.

The ABC measure correlates well with the raw bed net coverage but also deviates from it in many instances. Indeed, at three different levels different priority orders have been shown to arise when using the ABC. The ordering of countries and regions within countries changes when taking into account malaria endemicity. Moreover, at the level of sub-populations within countries, the existence or the absence of socioeconomic inequalities in the level of bed net coverage are not necessarily confirmed when using the ABC.

## Competing interests

The authors declare that they have no competing interests.

## Authors’ contributions

DV and NS conceived the study and drafted the manuscript. DV performed the analysis. Both authors read and approved the final manuscript.

## Supplementary Material

Additional file 1 Supporting text: Listing of the surveys and countries that were omitted from the study. Click here for file
