# Supplementary material for: An adjusted bed net coverage indicator with estimations for 23 African countries
Source: Malar J. 2013 Dec 20;12:457. doi: 10.1186/1475-2875-12-457 (PMC4021220; doi:10.1186/1475-2875-12-457)
Supplement: Additional file 1 — Supporting text: Listing of the surveys and countries that were omitted from the study. [file 1475-2875-12-457-S1.pdf]

Listing of the surveys and countries that were omitted from the study

Surveys dating from before 2000 were not used in the current study. Therefore, the following countries were omitted as the only available survey for them was deemed too old:

1. Comoros
2. The Central African Republic
3. South Africa
4. Sudan
5. Togo

For some countries no GPS data were available. In this case the country could not be included:

1. Congo Brazzaville
2. Niger
3. Sao Tome and Principe

Usually, the latest survey for a country was used. However, there are some exceptions:

1. Data for Kenya was from the 2008-09 survey. A later survey, Kenya 2010, is a IV/MCH SPA survey for which the data were not available at the time of this study not available.
2. Data for Mali from 2006 were used instead of data from 2010 as this latter survey was a DHS special survey.
3. Data for Namibia was from 2006-07. More recent data from Namibia in 2009 is from a HIV/MCH SPA survey for which data were not available.
4. The Ethiopia 2005 survey was used instead of the 2011 survey as this did not contain information on bed net use.

Some surveys had to be omitted as they did not contain the necessary variables:

1. Data from Chad was not used as the only survey containing information on bed net use in children was from before 2000.
2. Data from Lesotho was not used as neither the 2009 survey or the 2009 survey did not contain information on bed net use.
3. The surveys from Gabon and cote d'ivoire did not contain information on bed net use.
4. data from Benin could not be used as the 2006 survey lacked GPS data and the 2001 survey lacked information on bed net use.

finally, Swaziland not included because malaria not endemic in this country.
